# Optical coherence tomography angiography characteristics of optic disc melanocytoma

**DOI:** 10.1186/s12886-020-01676-7

**Published:** 2020-10-27

**Authors:** Nan Zhou, Xiaolin Xu, Wenbin Wei

**Affiliations:** Beijing Tongren Eye Center, Beijing Key Laboratory of Intraocular Tumor Diagnosis and Treatment, Medical Artificial Intelligence Research and Verification Laboratory of the Ministry of Industry and Information Technology, Beijing Tongren Hospital, Capital Medical University, Beijing, China

**Keywords:** Optical coherence tomography angiography, Pigmentation, Vascular density, Perfusion density, Optic disc melanocytoma

## Abstract

**Background:**

Optic disc melanocytoma (ODMC) was a benign pigmented intraocular tumor with the rare potential malignant transformation. This study was designed to evaluate tumor vasculature with optical coherence tomography angiography (OCTA) in ODMC.

**Methods:**

Eyes of healthy individuals in a control group and of patients with ODMC were imaged by OCTA systems operating at 840 nm wavelengths and examined. The main outcome measures were OCTA images, qualitative evaluation of optic disc and tumor vasculature, quantitative vascular density (VD) and perfusion density (PD).

**Results:**

One eye of ten normal volunteers and ten patients with ODMC were imaged. Eyes affected by ODMC as compared to the eyes of the control group (all *P* < 0.05). The healthy optic disc had radially-oriented vessels within the retina on OCTA. Optic disc melanocytoma was characterized by globular, demonstrated tortuous blood vessels, uneven thickness, and relatively disorganized intratumoral vasculature. The VD and PD within ODMC were significantly higher (12.360% ± 4.175, 0.316% ± 0.119%, *P* < 0.0001) than in normal optic discs (4.160% ± 2.290, 0.102% ± 0.0, 56%, *P* < 0.0001). No significant differences were established of the VD and PD in each single measurement zone (*P* > 0.05) between the ODMC and the control eyes. At 840 nm, OCTA could provide sufficient visualization of the tumor vasculature and better penetration through thicker tumors. The full thickness was visualized even in thicker tumors and highly pigmented lesions (> 2 mm). Interpretable OCTA images were obtained in 96% of the participants in whom imaging was attempted.

**Conclusions:**

OCTA may provide a noninvasive, safe, and efficient technique for evaluating a variety of neoplasms including the growth and vascularity in ODMC. OCTA could facilitate the evaluation of the vascular abnormalities of tumors and the effect of melanin on the penetration of the OCTA beam was not significant.

## Background

Optical coherence tomography angiography (OCTA) was a dye-free and relatively new microvascular imaging method that has been applied to evaluate retinopathies or choroidal neovascularization and the changes in different vascular layers [1, 2]. This technique does not require injected contrast agents, which increases its safety and decreases its risk compared with traditional ophthalmic angiography tests, such as indocyanine green angiography (ICGA) and fluorescein angiography (FA). The application of OCTA system to evaluate and analyze the tumor vascular system was a newly developing field [3, 4]. Previous studies have shown that the OCTA system working at 840 nm wavelength could improve the penetration into turbid media and decrease light scattering by tumor tissues [5, 6]. We used the Carl Zeiss Meditec OCTA system operating at 840 nm wavelength in our study to investigate optic disc pigmented tumors.

Optic disc melanocytoma (ODMC) was a benign pigmented hamartoma with rare malignant potential, but approximately 1–2% cases have been observed to acquire malignant transformation [7]. They may infiltrate the adjacent retina and choroid [8–10], and while they tend to be less aggressive than posterior choroidal melanoma. Most ODMC patients were asymptomatic with no visual loss [11]. Histopathologically, melanocytoma was composed of intensely pigmented round to oval nevus cells with benign features. However, slow growth was observed in approximately 11% of the cases [12]. Diagnosis was based on ophthalmoscopic features [7]. Ancillary tests such as ultrasonography and optical coherence tomography (OCT) helped in follow-up. Clinically, the increased tumor vascularity may be an indication associated with aggressive tumor behavior with metastatic potential [12]. OCTA used special algorithms to construct maps of blood flow in various tissue layers and further provided a way to generate quantitative indicators to analyze the state of the vascular system. OCTA could provide vascular changes associated with the malignant transformation of tumors.

The purpose of this preliminary study was to characterize and quantify the ODMC using OCTA, by comparing the vascular patterns, vascular density, and perfusion density in the ODMC. We also proposed OCTA imaging of normal blood flow of optic disc in healthy volunteers, which were utilized to evaluate the tumorigenesis-associated changes in vascularity.

## Methods

The clinical cross-sectional observational study included patients who consecutively recruited in the Beijing Tongren Eye Center, Beijing Tongren Hospital from April 2018 to January 2019. The healthy participants were volunteers.

All patients with ODMC underwent detailed ophthalmologic examination and imaging, including best-corrected visual acuity (BCVA), intraocular pressure (IOP), slit lamp biomicroscopy, indirect ophthalmoscope, fundus color photography, ICGA, and FA.

Based on the fundus examination, ODMC was characterized by brown to black pigmented mass mainly located at the optic disc, without other fundus diseases. Patients were excluded if they previously had undergone retinal surgery or showed signs of glaucoma, retinal vein occlusions, diabetic retinopathy, age-related macular degeneration, or other retinal diseases. The normal individuals with no history of any systematic disease or ocular disease served as the control groups, a BCVA of 20/20 or better. The age, gender, and laterality of the control groups matched to the ODMC patients.

The clinical examination and review of imaging were performed by an ophthalmic oncologist (W.W.B.) with rich experience in the clinical diagnosis of ODMC. All healthy volunteers were subjected to fundus color photography and OCTA, reviewed by an ophthalmologist (W.W.B.).

### OCTA

Each of the ODMC eyes and the healthy eyes of the control group was evaluated using AngioPlex device (Cirrus 5000 HD-OCT system, Carl Zeiss Meditec, Inc.) acquired OCT angiograms. Additional scans using the 20 Hz FAST-TRAC™ Technology, which provides live-tracking for motion-artifact-free images [13]. The AngioPlex Metrix™ technology provides the measure of vascular density and perfusion density images. The Single-Scan Simplicity of the ZEISS AngioPlex system requires only a single additional OCT scan to generate an ultra-clear three-dimensional (3D) OCTA image. Participants’ pupils were pharmacologically dilated to allow visualization of the fundus. Three-dimensional (3D) horizontal and vertical OCTA raster data was acquired over retina mode (3 × 3 mm, 6 × 6 mm) with a scan depth of 2 mm inside the tissue. Each OCTA raster scan took approximately 2.5 s to obtain three repeated B-scans. The artifacts caused by axial movement from eye motion was eliminated by post-imaging software. The software automatically segmented the tissue into six layers: the vitreoretinal interface (VRI), the superficial retinal vascular layer (SRL), the deep retinal vascular layer (DRL), the avascular, the choriocapillaris layer, and the choroid layer. The segmentation of the tissue layers was checked before any measurement was performed. For ODMC patients, we edited the layers of the tumors that may cause the algorithms to incorrectly trace the actual boundaries. In the cross-sectional OCT images, the surface of the ODMC and the borderline of the retinal pigment epithelium (RPE) were segmented.

The vascular density (VD, defined as the total length of perfused vasculature per unit area in a region of measurement) and perfusion density (PD, defined as the total area of the perfused vasculature per unit area in a region of measurement) were used as vascular quantitative indicators in AngioPlex Metrix™ OCTA system [14]. Using the optical microangiography (OMAGC) algorithm to generate the OCTA images and to measure the VD and PD in ODMC and healthy optic disc region. For the SRL, the VD and PD were calculated and separately in nine regions (central, inner superior, inner inferior, inner temporal, inner nasal, outer superior, outer inferior, outer temporal and outer nasal), and the total area. The congruency between both methods was evaluated. We reviewed both 3 × 3-mm and 6 × 6-mm scans centered on the optic disc in all subject eyes.

### Statistical analysis

Student’s *t*-test was used to compare the PD and VD measurements between ODMC and the normal optic disc region. Statistical analysis was then performed through a statistical software package (SPSS for Mac, version 25.0; IBM-SPSS, Chicago, IL, USA). The data of the measurements were compared with each other using the paired Student’s *t*-test. All measurements were described as mean ± standard error. All two-sided tests *P*-values < 0.05 were considered statistically significant.

## Results

**Fig. 1:**
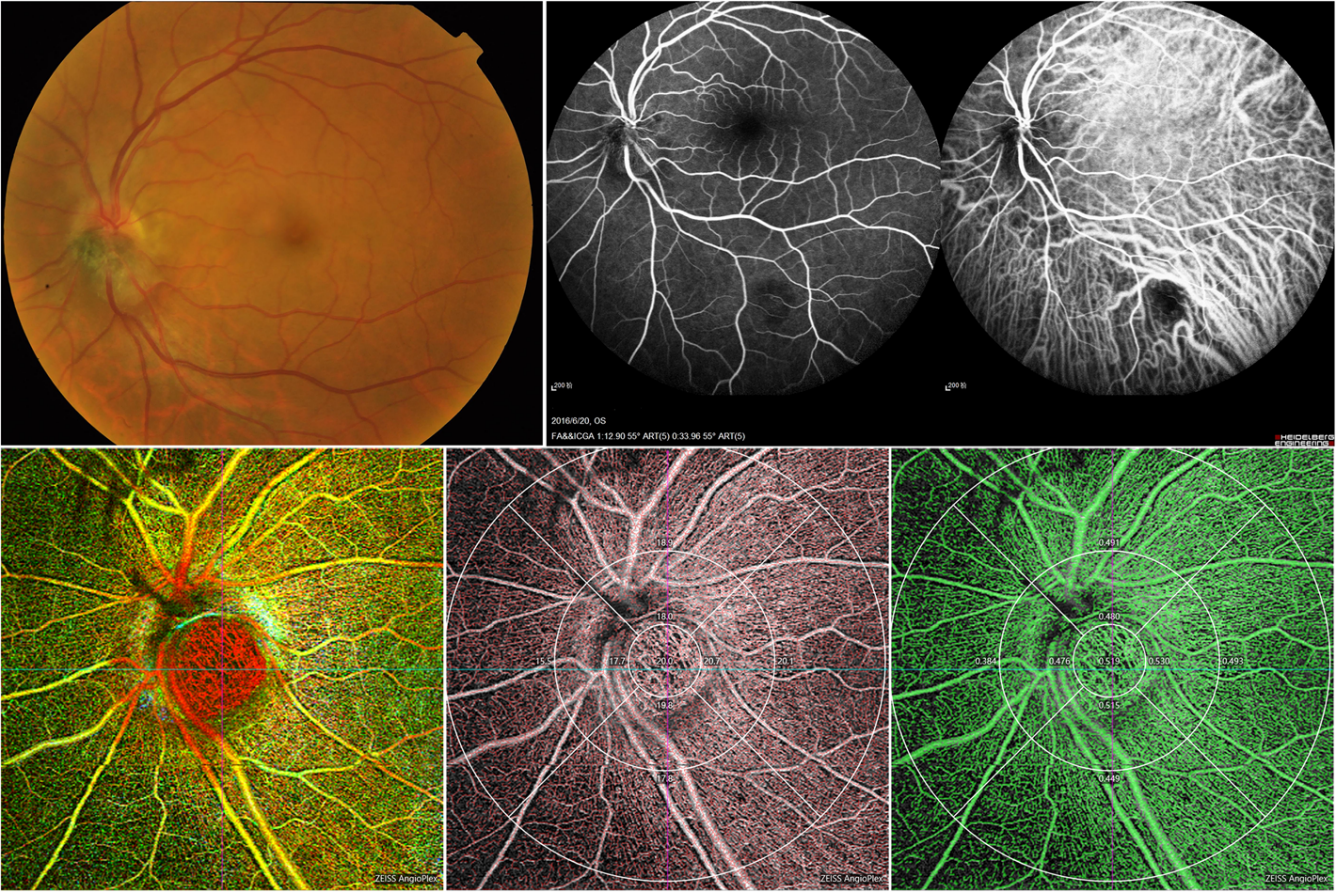
Retina Depth Encoded OCTA at 840 nm in affected eyes. Blood vessels within ODMC were characterized with predominantly heterogeneously distributed and disorganized intratumoral vasculature are visible (**c**), which is not consistent with the previously described fundus photograph (**a**), fluorescein angiography (FA) and ICGA appearance (**b**) of the ODMC vasculature. There was good penetration of the flow signal in the ODMC and normal optic disc tissue areas in OCTA images. The vascular density (**d**) and perfusion density (**e**) within ODMC was measured by AngioPlex MetrixTM OCTA images. Nine main regions were evaluated for the purposes of this study (6 × 6-mm)

Ten eyes of ten healthy participants (six male, four females; mean age 46.70 ± 10.83 years, range 25 to 58 years) and ten eyes of ten patients (six male, four females; mean age 46.70 ± 10.83, range 25–58 years) with ODMC were prospectively included in this observational study. The demographic information of the enrolled participants was presented in Table 1. Ophthalmoscopic examination revealed dark brown or black solid dome-shaped masses with well-defined margins in all 10 patients. Retina Depth Encoded OCTA clearly visualized blood vessels in the ODMC with preponderantly heterogeneously distributed and relatively disorganized intratumoral vasculature, which was not consistent with the previously described FA / ICGA appearance of ODMC vascular system (Fig. 1). There was good penetration of blood flow signal in the ODMC and normal optic disc tissue areas in OCTA (6 mm × 6 mm). However, the visualization of the vessels in the dome-shaped elevation of melanocytoma and in the most pigmented portion of the tumor was not impeded by OCT signal (Fig. 1).

**Table 1:** Demographic characteristics and quantitative analysis of vascular density between ODMCs and controls

	Controls (*n* = 10)	ODMCs (*n* = 10)	*P* Value
Sex (male/female)	6/4	6/4	–
Age (years)	46.70 ± 10.83	46.70 ± 10.83	–
Eye (right/left)	4/6	6/4	–
Total vascular density (%)	144.950 ± 19.638	146.620 ± 22.036	0.860
Optic disk vascular density (%)	4.160 ± 2.290	12.360 ± 4.175	<0.0001
Inner ring vascular density (%)			
Temporal	17.090 ± 2.610	18.670 ± 4.718	0.366
Superior	16.480 ± 3.362	18.350 ± 2.484	0.174
Nasal	17.520 ± 3.660	16.680 ± 2.756	0.569
Inferior	17.750 ± 1.968	16.200 ± 4.400	0.323
Outer ring vascular density (%)			
Temporal	16.860 ± 2.927	17.060 ± 4.846	0.912
Superior	18.740 ± 2.054	17.210 ± 2.800	0.181
Nasal	17.860 ± 4.432	14.210 ± 4.821	0.095
Inferior	18.490 ± 2.093	15.880 ± 4.232	0.097

Data were presented as mean ± SD; statistical analyses were Student’s t-test

For the 10 ODMC eyes, OCTA imaging had been performed. OCTA imaging had a better visualization of benign ODMC. The OCTA of ODMC revealed the increased vascularity and vasculature within the tumor was heterogeneously distributed and densely, with rich and brush-like tumor blood vessels, uneven thickness, and few small vascular loops were formed (Fig. 1). The areas of attenuated signals might have indicated the absence of blood vessels or the presence of extraordinary densely packed red blood cells.

In contrast, OCTA provided sufficient penetration, the tumor vasculature was not masked by the pigmentation in the tumor in the 840 nm OCTA, even in cases of highly pigmented lesions. OCTAs (Fig. 1) showed very dense and relatively organized intratumoral vessels. In the highly pigmented melanocytoma, cross-sectional OCTA showed that the tumor contains abundant blood vessels and most vessels were located in the tumor. In the case of variably pigmented, there was still extremely dense vascularization with slight shadowing below the vessels. In this study, OCTA could visualize the full thickness of pigmented ODMC in the range of 0.72–2.13 mm (as measured by OCT).

**Fig. 2:**
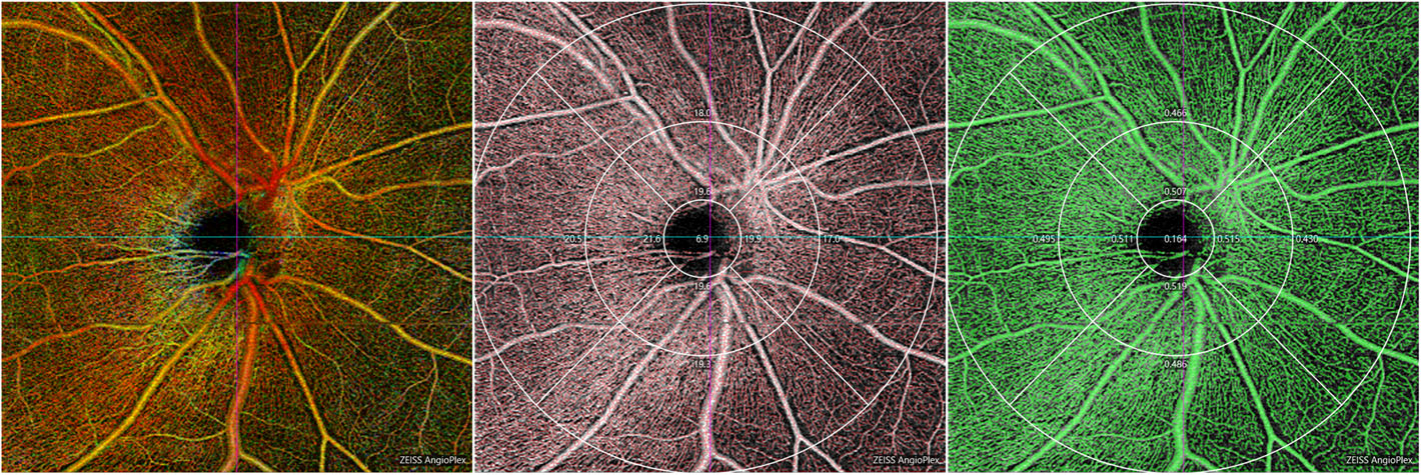
Vascular density and perfusion density in the normal optic disc, measured using AngioPlex MetrixTM OCTA images. Nine main regions were evaluated for the purposes of this study. The radial peripapillary capillary network was visualized in scans centered on the optic nerve head (ONH) by performing a 6 × 6-mm scan over the nerve (**a**). The vascular density (**d**); perfusion density (**e**)

In the normal eyes, the radial peripapillary capillary network can be seen in scans centered on the optic nerve head (ONH) by acquiring 6 × 6 mm scan mode. The images of the radial peripapillary capillary network were merged through elastic warping of the images, the larger vessels appeared blue color and the radial peripapillary capillaries red color (Fig. 2). The VD and PD within ODMC and in the normal optic disc areas were measured and quantified using AngioPlex MetrixTM OCTA images.

**Fig. 3:**
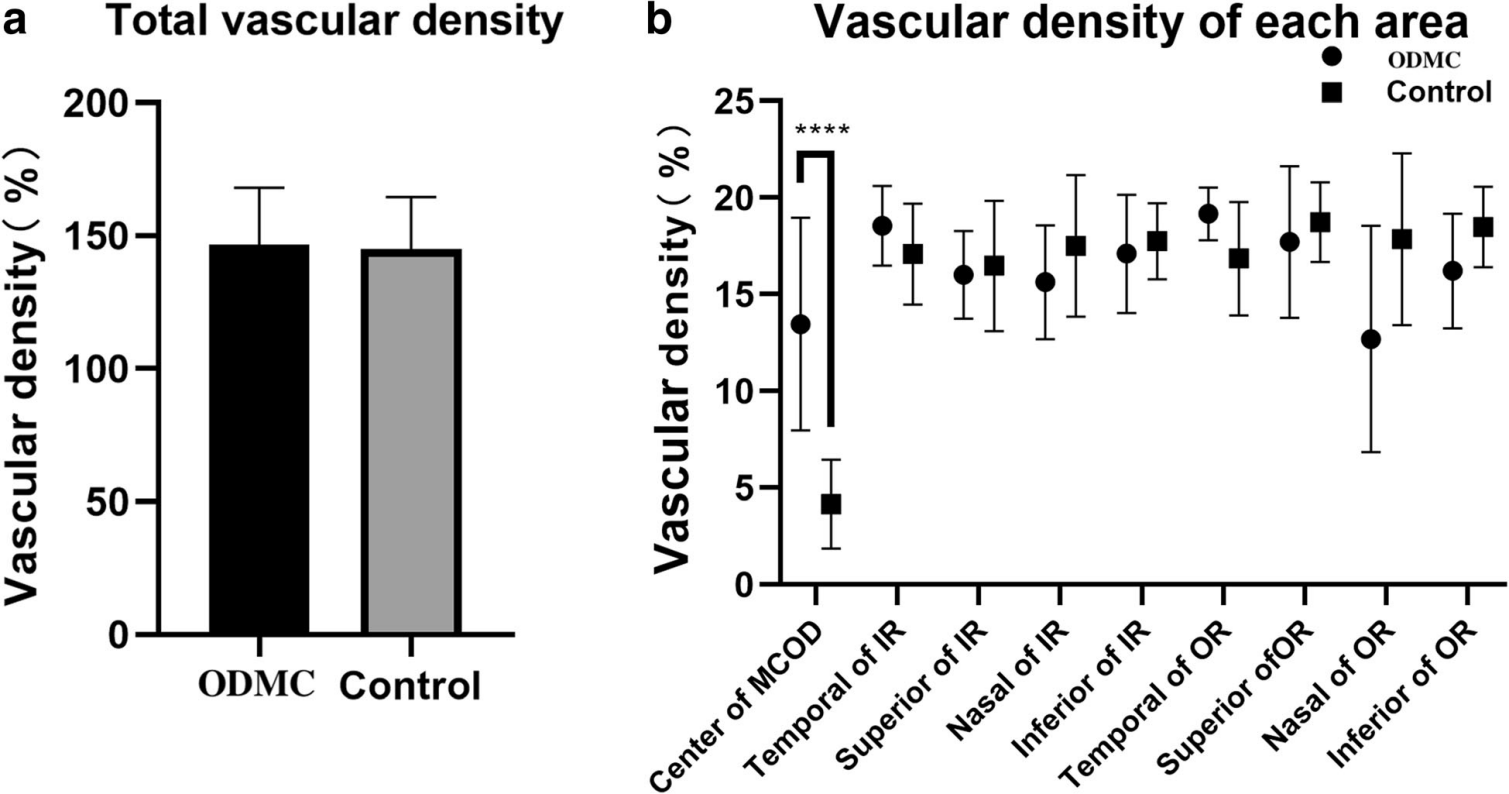
Vascular density comparisons in the control group and ODMC groups in areas centered on the optic disk. **a** Vascular density comparison of the total areas; **b** Comparison of the vascular density between the control group and ODMC groups in 9 single areas.****compared with the previous stage, *P* < 0.0001. IR, inner ring; OR, outer ring

**Fig. 4:**
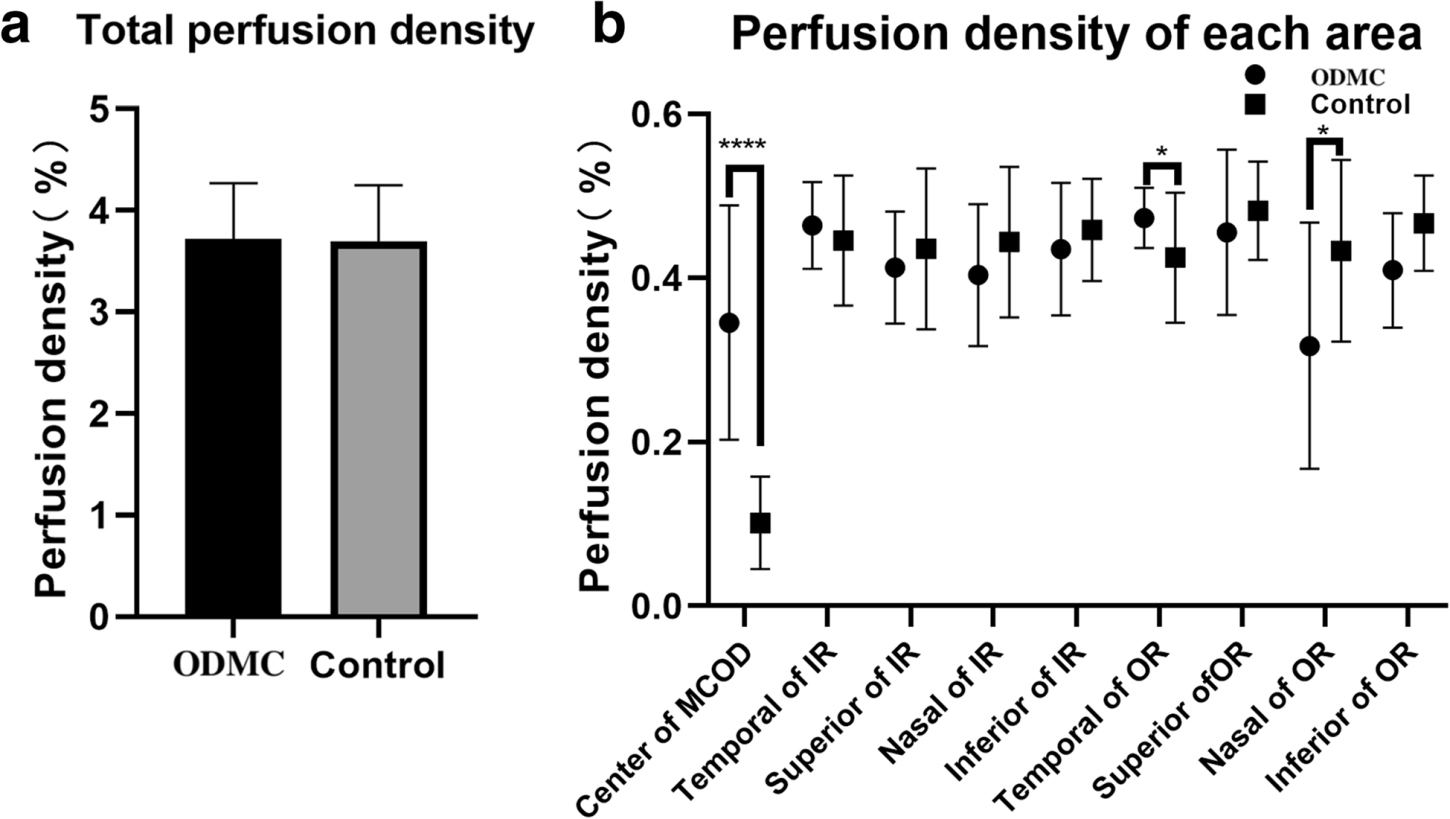
Perfusion density comparisons in the control group and ODMC group in areas centered in the optic disk. **a** Perfusion density comparison of the total areas; (**b**) Comparison of the perfusion density between the control group and ODMC groups in 9 single areas.****compared with the previous stage, *P* < 0.0001. IR, inner ring; OR, outer ring

The total area and the nine major areas were evaluated for this study. Compared with the healthy optic discs, OCTA of ODMC showed increased blood vessels associated with the tumor lesions. The VD and PD were significantly higher in ten ODMC (12.360% ± 4.175, 0.316% ± 0.119%, *P* < 0.0001) than in the normal optic discs (4.160% ± 2.290, 0.102% ± 0.0, 56%, *P* < 0.0001) (Figs. 3, 4). There was no significant difference in VD and PD between ODMC and control eyes in every single region (*P* > 0.05) (Tables 1, 2), whose quantification of blood vessels was with adequate repeatability and reproducibility using optical microangiography (OMAG)-based OCTA.

**Table 2:** Quantitative analysis of perfusion density between ODMCs and controls

	Controls (*n* = 10)	ODMCs (*n* = 10)	*P* Value
Total perfusion density	3.670 ± 0.547	146.570 ± 21.491	0.925
Optic disk vascular density (%)	0.102 ± 0.056	0.316 ± 0.119	<0.0001
Inner ring vascular density (%)			
Temporal	0.446 ± 0.080	0.454 ± 0.113	0.857
Superior	0.436 ± 0.098	0.471 ± 0.064	0.352
Nasal	0.444 ± 0.092	0.419 ± 0.060	0.470
Inferior	0.459 ± 0.063	0.398 ± 0.106	0.136
Outer ring vascular density (%)			
Temporal	0.425 ± 0.079	0.420 ± 0.122	0.908
Superior	0.482 ± 0.060	0.426 ± 0.079	0.090
Nasal	0.433 ± 0.111	0.352 ± 0.121	0.133
Inferior	0.467 ± 0.058	0.415 ± 0.103	0.178

Data were presented as mean ± SD; statistical analyses were Student’s t-test

## Discussion

OCTA provided a unique and non-invasive technique for the evaluation of blood flow and vascular density and has been applied to the prediction of disease beyond the retinal vascular disease, such as Alzheimer’s disease and Parkinson’s disease [15, 16]. The imaging study of intraocular tumors using OCTA was a new field. In our study, we discovered that the OCTA system could be applied to image ODMC and quantify tumor vascularity.

The formation of solid tumors would undergo the initial avascular phase and the subsequent angiogenic phase. Neovascularization was a sign of malignant transformation [17–19]. The increase of intratumoral microvessel density was related to the increase of tumor invasiveness and aggressiveness, which was a morphological indicator of angiogenesis [20]. It was noteworthy that OCTA may provide a non-invasive method to detect microvessel density in tumors and has potential applications in imaging a variety of ocular tumors and systemic tumors.

The OCT features of ODMC have been described as a dome-shaped mass with hyper-reflectivity of anterior surface and densely posterior shadowing with an optical cavity appearance [21]. It has been reported that 4–13% of the cases had vitreous pigment dissemination, but no malignant tumor cells [11, 22]. However, OCT did not show the intrinsic features of the tumor.

On the FA/ICGA, the ODMC showed an avascular tumor with hypofluorescence throughout all the phases, due to the blocked fluorescence by the pigmented tumor mass [12, 23]. FA/ICGA and OCT or their combination may not be able to reflect sufficiently tumor blood vessels well. Due to the better penetration, OCTA visualized the tumor vasculature would be unaffected by the pigmentation for it utilized the movement of red blood cells against stationary tissue as intrinsic contrast. In this study, OCTA revealed the presence of heterogeneously arranged small blood vessels and relatively disorganized intratumoral vasculature within the tumor, and the vascular structure was visible. ODMC was characterized by hypervascularity, with small densely tortuous vascular patterns and relatively organized arrangement and increased vascular density. The detection of vascularization of superficial tumors by OCTA has also been described in previous studies [24].

In our study, eyes affected by the ODMC group had higher VD and PD than those of the control group (*P* < 0.0001). Our present results were consistent with those of previous studies, which also evaluated retinal vascular changes related to intraocular tumors [25–27]. Interestingly, the VD and PD in each single measurement zone of the nine major regions showed no significant differences (*P* > 0.05) between the ODMC and the control eyes, maybe due to the tumor tissues were distributed differently in the single area of the measured region. Tumorigenesis and tumor regression was associated with the changes of the vessel density. The quantitative changes in vascular density and blood perfusion examined using OCTA may provide valuable information on tumorigenesis and signs of tumor regression.

Although ODMC generally was believed to have limited local complications, a recent study showed approximately 3% incidence of retinal vascular occlusions secondary to ODMC which could cause severe visual loss and usually was due to ischemic necrosis in the tumor or vascular compression by the tumor [12]. OCTA could also be helpful in detecting the changes in vascular parameters due to vascular occlusions, such as capillary drop out and/or telangiectasias with probable vascular dilation. These changes might affect intrinsic tumor vascularity but did not necessarily mean a malignant transformation.

ODMC patients were often asymptomatic and had good vision, but it was difficult to differentiate ODMC from early optic disc melanoma in morphology [28]. As there can be considerable morbidity associated with excisional biopsy or plaque radiotherapy for the treatment of melanoma of the optic disc, and the observation of lesions was associated with a risk for metastatic disease, developing a non-invasive method facilitating the prediction of tumor behavior was highly desirable. OCTA may provide a convenient and economical method for monitoring the optic disc tumors and helping to identify the lesions with the high-risk for malignant transformation and the potential of metastatic spread and diffusion. Therefore, OCTA may serve as a non-invasive tool to determine tumor vasculature at different tumor grades, and if there was tumor growth, close follow-up was needed to confirm the malignant transformation. This technique may also contribute to the avoidance of unnecessary local resections, biopsies, enucleations, or radiotherapy. Further study was needed to demonstrate the clinical application of this method.

Our study had several limitations. First, the size of the OCTA image could not sufficiently enough to capture the entire field of the retina in one scan. In this study, we acquired both 6 × 6 mm and 3 × 3 mm retinal mode containing the same axial scanning number. We found that the 6 × 6 mm scan was preferable as it provided better vascular details. Secondly, an important limitation of OCTA for imaging tumors was the inadequate penetration through higher/thicker tumors. Although not all ODMC can be imaged completely, 840 nm OCTA system was superior in the penetration of ONH neoplasms, so that vascular density and volume can be measured. Thirdly, the sample of our study was small, and further study with a large sample was needed to collect the more features and changes of tumor blood vessels.

## Conclusion

In summary, this study was the systematic investigation of applying OCTA in ODMC. The OCTA system successfully imaged the blood vessels within highly pigmented ODMC. This technique may be used as a noninvasive alternative to assess tumor vasculature at different layers, and it can also be used to quantitatively measure the VD and PD of intraocular tumors. Therefore, OCTA findings may serve as a biomarker to evaluate the tumor blood vessel patterns and help to monitor and judge the response to treatment.

## Data Availability

The data of this original article are available from the corresponding author on reasonable request.

## Abbreviations

OCTA: Optical coherence tomography angiography
ODMC: Optic disc melanocytoma
ICGA: Indocyanine green angiography
FA: Fluorescein angiography
ONH: Optic nerve head
VD: Vascular density
PD: Perfusion density
